# Sodium and Potassium Intake of Urban Dwellers: Nothing Changed in Yazd, Iran

**Published:** 2014-03

**Authors:** Masoud Mirzaei, Mohammadhossien Soltani, Mahdieh Namayandeh, Neda GharahiGhehi

**Affiliations:** ^1^Yazd Cardiovascular Research Centre, Shahid Sadoughi University, Yazd, Iran and Menzies Centre for Health Policy, University of Sydney, Sydney, Australia; ^2^Yazd Cardiovascular Research Centre, Shahid Sadoughi University, Yazd, Iran; ^3^Department of Epidemiology, Shiraz University of Medical Sciences, Shiraz, Iran; ^4^General Practitioner, Tehran, Iran

**Keywords:** 24-hour urine, Blood pressure, Cardiovascular disease, Hypertension, Policy, Potassium, Prevention, Salt, Sodium, Iran

## Abstract

To assess the daily salt intake of people aged 20-74 years based on the 24-hour urinary sodium excretion in urban population of Yazd, a population-based cross-sectional study was conducted. This is a substudy of Yazd Healthy Heart Project in Iran. From 2004 to 2005, two thousand people of the urban population of Yazd city, aged 20-74 years, were enrolled in the main study. Overall, 219 volunteer participants of 20-70 years were enrolled in this substudy. Sample frame was the household numbers according to the database of Yazd City Health Services. Calcium, phosphorus, sodium, potassium, and creatinine were measured in the urine samples collected from the participants over a 24-hour period. Sodium content in urine over 24 hours was 171.7±82.9 mmol/day in males and 127.8±56.1 mmol/day in females (p<0.0001) while potassium content was 49.4±23.2 mmol/day in males and 41.5±25.1 mmol/day in females (p=0.2). Estimated average daily salt (NaCl) intake was 10.0±4.8 g/day in males and 7.5±3.3 g/day in females (p<0.0001). Only one participant had the ideal Na/K ratio of less than one. Na/K ratios greater than one and less than two were seen in 11.3% (n=24), and a ratio equal to or greater than 2 was observed in 82.3% (n=118) of the participants. The average Na/K ratio was 3.69±1.58. Unlike many developed countries where sodium intake declined over the past few decades, the daily sodium intake in Yazd is high, and daily potassium intake is low. This is similar to what was observed four decades ago in an area not far from Yazd. Efforts must be directed towards health promotion interventions to increase public awareness to reduce sodium intake and increase potassium intake.

## INTRODUCTION

High blood pressure is one of the major established risk factors of cardiovascular and cerebrovascular diseases, including coronary heart disease and stroke. Sodium ion from salt (NaCl) has been proven to be a major determinant of high blood pressure (1).

Genetical susceptibility, dietary habits, environment, and lifestyle are the most important predictors of sodium intake (2). There is a strong body of evidence that supports the association between daily sodium intake in the form of salt consumption and hypertension (1,3,4). Furthermore, high sodium intake increases left ventricular muscle mass, wall thickness of vessels and increases the rate of stroke and heart failure, independent of hypertension (4).

The Intersalt Study investigated the relationship between sodium and hypertension in 52 people in 32 countries. The study showed a strong positive relationship between systolic and diastolic blood pressure and urinary sodium excretion (5).

Primary prevention is a vital long-term goal of public health. There are substantial benefits from even modest reductions of blood pressure from reducing salt intake (6). Dietary intervention trials demonstrated a significant result from reducing salt intake and increasing consumption of fruit and vegetables and non-dairy products. Additionally, a pilot programme in Nigeria has shown that good adherence to reducing sodium correlated with modest lowering of blood pressure despite the low baseline level (7).

Moreover, multiple interventional studies have demonstrated that reducing daily salt intake can reduce blood pressure (8). Evidence from randomized controlled trials, such as the DASH study, showed that reduction in sodium intake can decrease blood pressure in hypertensive individuals and, to a lesser extent, in normotensives. Limiting sodium intake to 67 mmol/day can reduce systolic and diastolic blood pressure by 5.8 and 2.5 mmHg respectively (3).

Reduction in sodium intake can be an effective public health intervention at the population level. A 2 mmHg reduction of mean blood pressure in general population can reduce mortality from cardiovascular diseases by 17% (9). Evidence shows that a 3 g/day decrease in the daily salt intake resulted in 13% decline in stroke-related mortality and 10% decline in ischaemic heart disease-related mortality (10).

Although the relationship between salt intake and blood pressure is linear, i.e. the lesser the intake the better, a general recommendation is to limit salt intake to 4.5-6 g/day (10). Eating more fresh fruits and vegetables and homemade foods instead of canned foods and fast foods are the core recommendations to reduce sodium intake (11).

The prevalence of hypertension according to the new criteria (>140/90 mmHg) varies across different regions of Iran between 15% and 35% in urban adult populations (12-14). This is similar to the prevalence of disease in other parts of Asia (15).

Recent hospital-based studies from Iran reported high consumption of salty food by almost 25% of patients with ischaemic heart disease in urban areas (16). After the study published by Page *et al.* in 1981 (17), little is known about the sodium and potassium intake in central Iran at the population level based on the objective method of 24-hour urine collection. This study aims to assess the daily salt intake by people aged 20-74 years according to the 24-hour urinary sodium execration in urban population of Yazd in central Iran.

## MATERIALS AND METHODS

This observational study was a substudy of Yazd Healthy Heart Project. From autumn 2004 to summer 2005, two thousand people from the urban population of Yazd city, aged 20-74 years, were enrolled in the main study based on cluster random sampling. Sample frame was the household numbers according to the database of Yazd City Health Services.

Yazd is located in central Iran, with over one million population. Sample-size was estimated according to the prevalence of high salt intake (18). To reach a power of 80% and detect the effect of a 25% decrease in mean salt intake (19), a sample-size of 220 was calculated. Overall, 219 voluntary participants aged 20-70 years, who agreed to accumulate their 24-hour urine were enrolled in this substudy. Enrolled participants were divided into five age-groups: 20-34, 35-44, 45-54, 55-64, and 65-74 completed years.

Participants were asked to collect all their urine outputs, except the one in early morning, in a given container and deliver it to the research centre the day after. The urine container contained 15 mL HCl (6 molar concentration). Biochemical analyses were performed at the Yazd Central Laboratory, using Corning 480 flame photometry (Tewksbury, MA, USA).

Out of 219 participants, six (2.7%) were excluded. We excluded samples with 24-hour urine volume equal to or greater than the value (weight x 15) for males and (weight x 10) for females (n=3) (20). Also, two samples were excluded because of high potassium contents and one for very low sodium content (20). Hypercalciuria (Ca >300 mg/day) was considered an indicator of high sodium intake (21). High sodium intake directly leads to increase in calcium excretion (22).

Calcium, phosphorus, sodium, potassium, and creatinine were measured in the 24-hour urine samples. The ions were reported in mmol/L of urine, multiplied by 24-hour urine volume and reported as mmol/day.

Daily urine volume of 0.5-1 mL/kg/hr was considered adequate hydration (23). To check the validity of test results, we randomly measured urine contents of five samples twice. Paired sample *t*-test indicated no significant difference.

Salt intake in dietary guidelines recommended g/day as unit; thus, we converted mmol/L to g/day. The mean daily salt intake was estimated by the following equation: (Na (mmol/day) x 58.5)/1,000=NaCl (g/day) (24).

In 24-hour urine collection, low potassium and high sodium contents may represent high sodium and low potassium intake in the study population. Thus, mean Na/K ratio was reported.

Ethical approval was obtained from the Ethics Committee of Shahid Sadoughi University. All participants signed informed consents to participate in the study.

To compare average of Na and K in men and women, independent sample *t*-test was used. To test difference between means of other variables, one-way ANOVA was used. All statistical analyses were carried out using SPSS software (version 12) (SPSS Inc, IL, USA).

## RESULTS

Table 1 demonstrates the characteristics of participants. The mean age of participants was 43.3±1 years for males and 43.5±1 years for females. Majority of participants (64.8 %) were male.

**Table 1.** Characteristics of the study participants

Variable	Prevalence of conditions	
	Yes No. (%)	No No. (%)
Sex		
Male	-	142 (64.8)
Female	77 (35.2)	
Current smoker	31 (14.6)	188 (85.4)
Diabetes mellitus	35 (16.4)	184 (83.6)
hypertension	84 (39.4)	135 (60.6)
High LDL	41 (19.2)	148 (80.8)
Abdominal obesity	57 (26.8)	162 (73.2)

**Table 2.** Average daily salt intake and sodium and potassium contents in 24-hour urine excretion among urban population of Yazd in 2005

Measure	Males 64.8% (n=138)	Females 35.2% (n=75)	p values#
Sodium*	171.67 (82.92)	127.78 (56.14)	<0.0001
Potassium*	49.36 (23.24)	41.54 (25.14)	0.2
Salt intake (NaCl)**	10.04 (4.85)	7.47 (3.28)	<0.0001

# For independent sample *t*-test;

*mmol/24-hr urine excretion;

**g/day; values are expressed as mean (SD)

**Table 3.** Average daily salt intake and 24-hour sodium and potassium excretion according to blood pressure status of urban population of Yazd in 2005

Measure	Normotensive 60.6% (n=129)	Diagnosed hypertension 18.3% (n=39)	Undiagnosed hypertension 21.1% (n=45)	p value$
Sodium*	145.76 (70.17)	150.63 (68.2)	185.87 (96.69)	<0.001
Potassium*	44.05 (24.66)	51.30 (21.66)	49.86 (24.27)	0.03
Salt (NaCl) intake	8.63 (4.1)	8.81 (3.98)	10.87 (5.65)	<0.001

$p value for one-way ANOVA;

*mmol/24-hour urine excretion;

values are expressed as mean (SD)

There was a significant difference (p<0.0001) in average daily salt intake of males (10.0±4.8 g/day) and females (7.5±3.3 g/day). The estimated average salt intake of the participants was 9.13 g/day, ranging from 1.17 g/day to 28.96 g/day (SD±4.52).

Based on urinary Na, males were eating more sodium and salt compared to females. Sodium contents in 24-hour urine samples was 171.7±82.9 mmol/day in males and 127.8±56.1 mmol/day in females (p<0.0001) while potassium content was 49.4±23.2 mmol/day in males and 41.5±25.1 mmol/day in females, (p=0.2). Table 2 shows average daily sodium and potassium excretion in 24-hour urine samples and estimated intake of salt (NaCl) for males and females.

Only one participant had the ideal Na/K ratio of less than one. Na/K ratios greater than one and less than 2 were seen in 11.3 % (n=24), and ratio equal to or greater than 2 was observed in 82.3% (n=118) of participants. The average Na/K ratio was 3.69±1.58.

Table 3 presents average daily salt intake and 24-hour sodium and potassium excretion according to blood pressure status of study participants.

Mean sodium excretion of undiagnosed hypertensive patients is significantly more than diagnosed patients (Table 3). Also, the mean potassium excretion of undiagnosed hypertensive patients was less than diagnosed patients (Table 3).

Estimated salt intake of normotensive population and diagnosed patients were not significantly different. However, undiagnosed patients had significantly higher estimated salt intake (Table 3).

Table 4 demonstrates the average 24-hour urinary sodium and potassium excretion by age-groups in urban population of Yazd. The measures are the highest in 45-54 years age-group (sodium=172.6±91.4 mmol/day and potassium=49.9±21.3 mmol/day) and the lowest in the 65-74 years age-group (sodium=123.8±73 mmol/day and potassium=35.6±20.3 mmol/day).

The figure shows percentage of participants with various daily sodium intakes in urban population of Yazd; 52% of the participants were in the 2.31-4.60 g/day group and around 5% in the more than 6.90 g/day group. Only 25% of participants can be considered optimal group according to the recommended daily sodium intake of less than 2.3 g/day (25).

Hypercalciuria (Ca>300 mg/day in men and >250 mg/day in women) was not reported in participant with less than 2.3 g/day sodium intake.

## DISCUSSION

Our study showed that daily sodium intake of a sample of urban dwellers of Yazd city in central Iran is over two times higher than the recommended level. It also demonstrated that the daily sodium urinary excretion was high in men and women, and the average Na/K ratio is almost 3.5 times more than the recommended ratio. The sodium intake was more in undiagnosed hypertensive patients.

In 1981, Page *et al.* (17) reported that 264 traditional nomadic herdsmen of the Qash'qai tribe in southern Iran. Members of the Qash'qai tribe, who were rural dwellers residing in a location 300 km from Yazd, had almost the same level of sodium intake and Na/K ratio some 40 years ago (17). Urinary sodium excretion averaged 186 mmol/day in males and 141 mmol/day in females. Urinary Na/K ratios were 3.64 and 3.24 in males and females respectively (17).

**Table 4.** Average sodium and potassium in 24-hour urine excretion*, by age-group, of urban population of Yazd in 2005

Age-group (completed years)	20-34 27.2% (n=58)	35-44 27.7% (n=59)	45-54 19.7% (n=42)	55-64 18.8% (n=40)	65-74 6.6% (n=14)
Sodium	150.02 (68.4)	169.1 (79.31)	172.55 (91.4)	140.4 (67.26)	123.84 (73.02)
Potassium	41.76 (24.1)	48.96 (23.79)	49.85 (21.3)	49.19 (28.08)	35.59 (20.3)

*mmol/24-hour urine excretion;

values are expressed as mean (SD)

**Figure.**
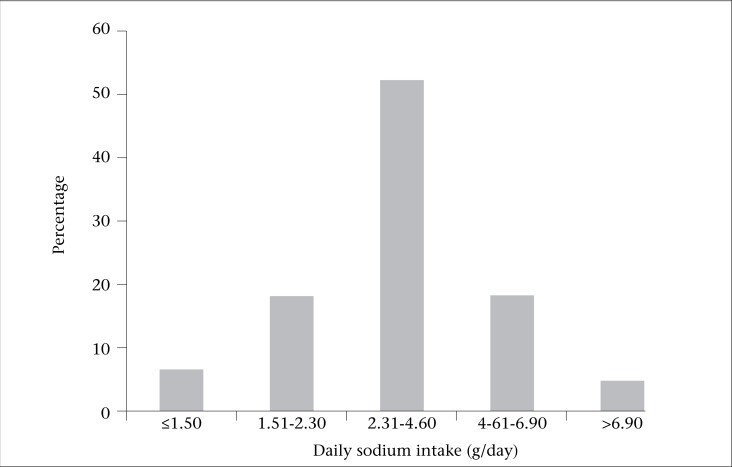
Daily sodium intake of urban population of Yazd in 2005

The study participants of Page *et al.* were slightly younger than ours, with age over 14 years compared to over 20 years in this study. The method of urine collection was similar to this study, with the exception that they used formaldehyde for preservation of samples. In 1976, after the oil boom, the process of industrialization had been extended to the rural and urban areas of Iran (17). Over the past four decades, access to healthcare and refrigerators has been remarkably increased in both areas.

In contrast, mean standardized 24-hour sodium excretion decreased from 265 to 188 mmol/day from 1967 to 1986 in Belgium. The decrease in stroke-related mortality in Belgium was due to combined effects of treatment for hypertension and a decrease in sodium intake (26).

The reduction of stroke-related mortality in Japan was much greater than in any other country of the world starting from 1965. Along with phenomenal effect of high blood pressure control, there are reports of a large reduction in NaCl intake in Japan. Salt intake in Japan reduced from 360 mmol/day in 1950 to 187 mmol/day in 1988 (27). These two examples demonstrate how community interventions can reduce salt intake at the population level in a few decades.

Our study, like another study (28), showed that the daily urinary sodium excretion is about 40-50 mmol/day less in women than in men. This difference is possibly related to more calorie intake in men as well as taking more fast foods, canned foods, and bread.

In Hobart salt study, the daily urinary potassium excretion was significantly more compared to that in our study (28). This difference may be related to different dietary regimens.

The average Na/K ratio was found high in this study (3.7±1.6). All the natural foods available to mammals have molar sodium to potassium ratio less than one. The human body must have been evolved on this dietary ratio, and its inversion is unnecessary and probably unsafe (29).

Hypercalciuria was not reported in participant with less than 2.3 g/day sodium intake. Hypercalciuria is an indicator of high sodium excretion (21).

In undiagnosed hypertensive patients, the daily salt intake is significantly higher than known hypertensive or normal persons according to our study. This finding suggests the positive relationship between high salt intake and hypertension and probably demonstrates the effect of medical advices from medical practitioners.

A systematic review of cardiovascular risk factors in the Middle East found that hypertension is highly prevalent in the population; overall prevalence of hypertension was 21.7% in the region (30).

The lowest age-standardized incidence rate of stroke in the Middle East was in Saudi Arabia (38.5 per 100,000) and the highest in Qatar (123.7 per 100,000) (31). Benner *et al.* suggested that the high incidence of stroke in Qatar could be due to eating, drinking, and other behaviours of the Qatari population (32).

A study on population-attributable fraction of haemorrhagic and ischaemic stroke relating to hypertension in men and women in the Middle East demonstrated that, in Iran, 41.5% of haemorrhagic stroke in men and 24% in women can be reduced by reducing blood pressure to the normal range among the average population. The reduction in ischaemic stroke, if the population blood pressure could be reduced to normal level, was reported to be 22% in men and 20.5% in women respectively (33).

Public awareness of hypertension and its prevention through simple means of diet and exercise may decrease the incidence of stroke in the population. The younger population in the Middle East, including Iran, indicates that as the years progress, the impact of stroke will increasingly become a major burden. Renewed emphasis on improved surveillance, prevention, and control of hypertension and stroke in Iran is much needed.

### Limitations

This study has some limitations. Bread is the main food in Iran, and the consumption of traditional ones is very high. Most traditional bakeries use sodium bicarbonate (baking soda) as leavening agent instead of yeast (34). In fact, the amount of measured sodium can be correct in this study but the amount of salt is likely to be overestimated.

### Conclusions

In urban area of Yazd, the daily sodium intake is high, and the daily potassium intake is low. This is similar to what was observed decades ago in an area not far from Yazd. Efforts must be directed towards health promotion interventions to increase public awareness to reduce sodium intake and increase potassium intake.

## ACKNOWLEDGEMENTS

Iranian Ministry of Health funded the study, with no influence on data analysis and interpretations. The authors acknowledge Professor A.S. Truswell for providing insightful comments, Associate Professor M. Shakiba for helping with nephrology aspects of the study, and Professor M. Sadr for conduction of the Yazd Healthy Heart Project.

## References

[1] Truswell AS (2010). Cholesterol and beyond: the research on diet and coronary heart disease, 1900-2000.

[2] Alderman MH (2002). Salt, blood pressure and health: a cautionary tale. Int J Epidemiol.

[3] Havas S, Roccella EJ, Lenfant C (2004). Reducing the public health burden from elevated blood pressure levels in the United States by lowering intake of dietary sodium. Am J Public Health.

[4] Meneton P, Jeunemaitre X, de Wardener HE, MacGregor GA (2005). Links between dietary salt intake, renal salt handling, blood pressure, and cardiovascular diseases. Physiol Rev.

[5] Dyer A, Elliott P, Chee D, Stamler J (1997). Urinary biochemical markers of dietary intake in the INTERSALT study. Am J Clin Nutr.

[6] Lawes CM, Bennett DA, Parag V, Woodward M, Whitlock G, Lam T (2003). Asia Pacific Cohort Studies Collaboration. Blood pressure indices and cardiovascular disease in the Asia Pacific region: a pooled analysis. Hypertension.

[7] Cooper RS, Amoah AG, Mensah GA (2003). High blood pressure: the foundation for epidemic cardiovascular disease in African populations. Ethn Dis.

[8] He FJ, MacGregor GA (2003). How far should salt intake be reduced. Hypertension.

[9] Lawes CM, Vander Hoorn S, Law MR, Elliott P, MacMahon S, Rodgers A (2006). Blood pressure and the global burden of disease 2000. Part II: estimates of attributable burden. J Hypertens.

[10] Frohlich ED, Varagic J (2005). Sodium directly impairs target organ function in hypertension. Curr Opin Cardiol.

[11] Misra A, Khurana L (2007). Salt intake and hypertension: walking the tight rope. J Assoc Physicians India.

[12] Bahrami H, Sadatsafavi M, Pourshams A, Kamangar F, Nouraei M, Semnani S (2006). Obesity and hypertension in an Iranian cohort study; Iranian women experience higher rates of obesity and hypertension than American women. BMC Public Health.

[13] Iran (2005). Ministry of Health and Medical Education. A national profile of NCD risk factors in I. R. Iran: selected results of the first survey of Italian NCD risk factor surveillance system, 2005.

[14] Sarraf-Zadegan N, Boshtam M, Mostafavi S, Rafiei M (1999). Prevalence of hypertension and associated risk factors in Isfahan, Islamic Republic of Iran. East Mediterr Health J.

[15] Singh RB1, Suh IL, Singh VP, Chaithiraphan S, Laothavorn P, Sy R (2000). Hypertension and stroke in Asia: prevalence, control and strategies in developing countries for prevention. J Hum Hypertens.

[16] Taghaddosi M, Vali GR (2003). [Behavioral habits and ischemic heart disease in Kashan, 1995]. Feyz.

[17] Page LB, Vandevert DE, Nader K, Lubin NK, Page (1981). Blood pressure of Qash'qai pastoral nomads in Iran in relation to culture, diet, and body form. Am J Clin Nutr.

[18] Asaria P, Chisholm D, Mathers C, Ezzati M, Beaglehole R (2007). Chronic disease prevention: health effects and ﬁnancial costs of strategies to reduce salt intake and control tobacco use. Lancet.

[19] Fahimi S, Pharoah P (2012). Reducing salt intake in Iran: priorities and challenges. Arch Iran Med.

[20] Landry DW, Bazari H (2011). Approach to the patient with renal disease. Chapter 116. In: Goldman L Schafer AI editors Goldman's Cecil medicine. 24th ed..

[21] Nouvenne A, Meschi T, Prati B, Guerra A, Allegri F, Vezzoli G (2010). Effects of a low-salt diet on idiopathic hypercalciuria in calcium-oxalate stone formers: a 3-mo randomized controlled trial. Am J Clin Nutr.

[22] Kaplan NM (2006). Treatment of hypertension: drug therapy. In: Kaplan NM editor Kaplan's clinical hypertension. 9th ed..

[23] Zaloga GP, Hughes SS (1990). Oliguria in patients with normal renal function. Anesthesiology.

[24] Dickinson BD, Havas S (2007). Council on Science and Public Health, American Medical Association. Reducing the population burden of cardiovascular disease by reducing sodium intake a report of the council on science and public health. Arch Intern Med.

[25] Whelton PK, Appel LJ, Sacco RL, Anderson CAM, Antman EM, Campbell N (2012). Sodium, blood pressure, and cardiovascular disease further evidence supporting the american heart association sodium reduction recommendations. Circulation.

[26] Joossens JV, Kesteloot H (1991). Trends in systolic blood pressure, 24-hour sodium excretion, and stroke mortality in the elderly in Belgium. Am J Med.

[27] Kesteloot H, Joossens JV (1992). Nutrition and international patterns of disease. In Marmot M Elliott P editors Coronary heart disease epidemiology: from aetiology to public health.

[28] Beard TC, Woodward DR, Ball PJ, Hornsby H, von Witt RJ, Dwyer T (1997). The Hobart Salt Study 1995: few meet national sodium intake target. Med J Aust.

[29] Yang Q, Liu T, Kuklina EV, Flanders WD, Hong Y, Gillespie C (2011). Sodium and potassium intake and mortality among us adults: prospective data from The Third National Health and Nutrition Examination Survey. Arch Intern Med.

[30] Motlagh B, O'Donnell M, Yusuf S (2009). Prevalence of cardiovascular risk factors in the Middle East: a systematic review. Eur J Cardiovasc Prev Rehabil.

[31] Tran J, Mirzaei M, Anderson L, Leeder SR (2010). The epidemiology of stroke in the Middle East and North Africa. J Neurol Sci.

[32] Bener A, Al-Suwaidi J, Al-Jaber K, Al-Marri S, Dagash MH, Elbagi IE (2004). The prevalence of hypertension and its associated risk factors in a newly developed country. Saudi Med J.

[33] Tran J, Mirzaei M (2011). The population attributable fraction of stroke associated with high blood pressure in the Middle East and North Africa. J Neurol Sci.

[34] Talaei M, Mohammadifard N, Khaje M-R, Sarrafzadegan N, Sajjadi F, Alikhasi H (2013). Healthy bread initiative: methods, findings, and theories–Isfahan Healthy Heart Program. J Health Popul Nutr.

